# The Spirit of Node Replication

**DOI:** 10.1007/978-3-030-71995-1_18

**Published:** 2021-03-23

**Authors:** Delia Kesner, Loïc Peyrot, Daniel Ventura

**Affiliations:** 8 University of Oxford, Oxford, UK; 9 Sorbonne Université - LIP6, Paris, France; 10 Université de Paris, CNRS, IRIF, Paris, France; 11 Institut Universitaire de France, Paris, France; 12 Univ. Federal de Goiás, Goiânia, Brazil

## Abstract

We define and study a term calculus implementing higher-order node replication. It is used to specify two different (weak) evaluation strategies: call-by-name and fully lazy call-by-need, that are shown to be observationally equivalent by using type theoretical technical tools.

## 1 Introduction

Computation in the $\lambda$-calculus is based on higher-order substitution, a complex operation being able to erase and copy terms during evaluation. Several formalisms have been proposed to model higher-order substitution, going from explicit substitutions (ES) [1] (see a survey in [41]) and labeled systems [15] to pointer graphs [60] or optimal sharing graphs [49]. The model of copying behind each of these formalisms is not the same.

Indeed, suppose one wants to substitute all the free occurrences of some variable $x$ in a term $t$ by some term $u$. We can imagine at least four ways to do that. (1) A drastic solution is a one-shot substitution, called *non-linear* (or *full*) *substitution*, based on simultaneously replacing *all* the free occurrences of $x$ in $t$ by the whole term $u$. This notion is generally defined by induction on the structure of the term $t$. (2) A refined method substitutes *one* free occurrence of $x$ at a time, the so-called *linear* (or *partial*) *substitution*. This notion is generally defined by induction on the number of free occurrences of $x$ in the term $t$. An orthogonal approach can be taken by replicating *one* term-constructor of $u$ *at a time*, instead of replicating $u$ as a whole, called here *node replication*. This notion can be defined by induction on the structure of the term $u$, and also admits two versions: (3) non-linear, *i.e.* by simultaneously replacing all the occurrences of $x$ in $t$, or (4) linear. The linear version of the node replication approach can be formally defined by combining (2) and (3).

It is not surprising that different notions of substitution give rise to different evaluation strategies. Indeed, linear substitution is the common model in well-known abstract machines for call-by-name and call-by-value (see *e.g.* [3]), while (linear) node replication is used to implement fully lazy sharing [60]. However, node replication, originally introduced to implement optimal graph reduction in

⋆ Supported by CNPq grant Universal 430667/2016-7.

a graphical formalism, has only been studied from a Curry-Howard perspective by means of a term language known as the atomic $\lambda$-calculus [33].

**The Atomic Lambda-Calculus.** The Curry-Howard isomorphism uncovers a deep connection between logical systems and term calculi. It is then not surprising that different methods to implement substitution correspond to different ways to normalize logical proofs. Indeed, full substitution (1) can be explained in terms of natural deduction, while partial substitution (2) corresponds to cut elimination in Proof-Nets [2]. Replication of nodes (3)-(4) is based on a Curry-Howard interpretation of deep inference [32,33]. Indeed, the logical aspects of intuistionistic deep inference are captured by the atomic $\lambda$-calculus [33], where copying of terms proceeds *atomically*, *i.e.* node by node, similar to the optimal graph reduction of Lamping [49].

The atomic $\lambda$-calculus is based on *explicit control of resources* such as erasure and duplication. Its operational semantics explicitly handles the structural constructors of weakening and contraction, as in the calculus of resources $\lambda\texttt{lxr}$ [43,44]. As a result, comprehension of the meta-properties of the term-calculus, in a higher-level, and its application to concrete implementations of reduction strategies in programming languages, turn out to be quite difficult. In this paper, we take one step back, by studying the paradigm of *node replication* based on *implicit*, rather than *explicit*, weakening and contraction. This gives a new concise formulation of node replication which is simple enough to model different programming languages based on reduction strategies.

**Call-by-Name, Call-by-Value, Call-by-Need.** *Call-by-name* is used to implement programming languages in which arguments of functions are first copied, then evaluated. This is frequently expensive, and may be improved by *call-by-value*, in which arguments are evaluated first, then consumed. The difference can be illustrated by the term $t = \Delta(\texttt{II})$, where $\Delta = \lambda x.xx$ and $\texttt{I} = \lambda z.z$: call-by-name first duplicates the argument $\texttt{II}$, so that its evaluation is also duplicated, while call-by-value first reduces $\texttt{II}$ to (the value) $\texttt{I}$, so that duplications of the argument do not cause any duplicated evaluation. It is not always the best solution, though, because evaluating erasable arguments is useless.

*Call-by-need*, instead, takes the best of call-by-name and call-by-value: as in call-by-name, erasable arguments are not evaluated at all, and as in call-by-value, reduction of arguments occurs at most once. Furthermore, call-by-need implements a *demand-driven* evaluation, in which erasable arguments are never needed (so they are not evaluated), and non-erasable arguments are evaluated only if needed. Technically, some sharing mechanism is necessary, for example by extending the $\lambda$-calculus with explicit substitutions/let constructs [7]. Then $\beta$-reduction is decomposed in at least two steps: one creating an explicit (pending) substitution, and the other ones (linearly) substituting *values*. Thus for example, $(\lambda x.xx)(\texttt{II})$ reduces to $(xx)[x\backslash\texttt{II}]$, and the substitution argument is thus evaluated in order to find a value before performing the linear substitution.

Even when adopting this wise evaluation scheme, there are still some unnecessary copies of redexes: while only *values* (*i.e.* abstractions) are duplicated, they may contain redexes as subterms, *e.g.* $\lambda z.z(\mathtt{II})$ whose subterm $\mathtt{II}$ is a redex. Duplication of such values might cause redex duplications in *weak* (*i.e.* when evaluation is forbidden inside abstractions) call-by-need. This happens in particular in the *confluent* variant of weak reduction in [52].

**Full laziness.** Alas, it is not possible to keep all values shared forever, typically when they potentially contribute to the creation of a future $\beta$-reduction step. The key idea to gain in efficiency is then to keep the subterm $\mathtt{II}$ as a *shared* redex. Therefore, the (full) value $\lambda z.z(\mathtt{II})$ to be copied is split into two separate parts. The first one, called *skeleton*, contains the minimal information preserving the bound structure of the value, *i.e.* the linked structure between the binder and each of its (bound) variables. In our example, this is the term $\lambda z.zy$, where $y$ is a fresh variable. The second one is a multiset of *maximal free expressions* (MFE), representing all the shareable expressions (here only the term $\mathtt{II}$). Only the skeleton is then copied, while the problematic redex $\mathtt{II}$ remains shared:

$$(\lambda x.xx)(\lambda z.z(\mathtt{II})) \to (xx)[x\backslash\lambda z.z(\mathtt{II})] \to ((\lambda z.zy)x)[x\backslash\lambda z.zy][y\backslash\mathtt{II}]$$

When the subterm $\mathtt{II}$ is needed ahead, it is first reduced inside the ES, as it is usual in (standard) call-by-need, thus avoiding to compute the redex twice. This optimization is called *fully lazy sharing* and is due to Wadsworth [60].

In the confluent weak setting evoked earlier [52], the fully lazy optimization is even optimal in the sense of Lévy [51]. This means that the strategy reaches the weak normal form in the same number of $\beta$-steps as the shortest possible weak reduction sequence in the usual $\lambda$-calculus without sharing. Thus, fully lazy sharing turns out to be a *decidable* optimal strategy, in contrast to other weak evaluation strategies in the $\lambda$-calculus without sharing, which are also optimal but not decidable [11].

**Contributions.** The first contribution of this paper is a term calculus implementing (full) node replication and internally encoding skeleton extraction (Sec. 2). We study some of its main operational properties: termination of the substitution calculus, confluence, and its relation with the $\lambda$-calculus.

Our second contribution is the use of the node replication paradigm to give an alternative specification of two evaluation strategies usually described by means of full or linear substitution: call-by-name (Sec. 4.1) and weak fully lazy reduction (Sec. 4.2), based on the key notion of skeleton. The former can be related to (weak) head reduction, while the latter is a fully lazy version of (weak) call-by-need. In contrast to other implementations of fully lazy reduction relying on (external) meta-level definitions, our implementation is based on formal operations internally defined over the term syntax of the calculus.

Furthermore, while it is known that call-by-name and call-by-need specified by means of full/linear substitution are observationally equivalent [7], it was not clear at first whether the same property would hold in our case. Our third contribution is a proof of this result (Sec. 6) using semantical tools coming from proof theory –notably intersection types. This proof technique [42] considerably simplifies other approaches [7,54] based on syntactical tools. Moreover, the use of intersection types has another important consequence: standard call-by-name and call-by-need turn out to be observationally equivalent to call-by-name and call-by-need with node replication, as well as to the more semantical notion of neededness (see [45]).

Intersection types provide quantitative information about fully lazy evaluation so that a fourth contribution of this work is a measure based on type derivations which turns out to be an upper bound to the length of reduction sequences to normal forms in a fully lazy implementation.

More generally, our work bridges the gap between the Curry-Howard theoretical understanding of node replication and concrete implementations of fully lazy sharing. Related works are presented in the concluding Sec. 7.

## 2 A Calculus for Node Replication

We now present the syntax and operational semantics of the $\lambda\mathtt{R}$-calculus ($\mathtt{R}$ for $\mathtt{R}$eplication), as well as a notion of *level* playing a key role in the next sections.

**Syntax.** Given a countably infinite set $\mathcal{X}$ of variables $x, y, z, ...$, we consider the following grammars.

| | |
|---|---|
| **(Terms)** | $t, u ::= x \mid \lambda x.t \mid tu \mid t[x\backslash u] \mid t[x\backslash\!\!\backslash \lambda y.u]$ |
| **(Pure Terms)** | $p, q ::= x \mid \lambda x.p \mid pq$ |
| **(Term Contexts)** | $\mathtt{C} ::= \Box \mid \lambda x.\mathtt{C} \mid \mathtt{C}t \mid t\mathtt{C} \mid \mathtt{C}[x\backslash t] \mid \mathtt{C}[x\backslash\!\!\backslash \lambda y.u] \mid t[x\backslash \mathtt{C}] \mid t[x\backslash\!\!\backslash \lambda y.\mathtt{C}]$ |
| **(List Contexts)** | $\mathtt{L} ::= \Box \mid \mathtt{L}[x\backslash u] \mid \mathtt{L}[x\backslash\!\!\backslash \lambda y.u]$ |

The set of terms (resp. **pure** terms) is denoted by $\Lambda_{\mathtt{R}}$ (resp. $\Lambda$). We write $|t|$ for the **size** of $t$, *i.e.* for its number of constructors. We write $\mathtt{I}$ for the identity function $\lambda x.x$. The construction $[x\backslash u]$ is an **explicit substitution (ES)**, and $[x\backslash\!\!\backslash \lambda y.u]$ an **explicit distributor**: the first one is used to copy arbitrary terms, while the second one is used specifically to duplicate abstractions. We write $[x \triangleleft u]$ to denote an **explicit cut** in general, which is either $[x\backslash u]$ or $[x\backslash\!\!\backslash u]$ when $u$ is $\lambda y.u'$, typically to factorize some definitions and proofs where they behave similarly in both cases. When using the general notation $t[x \triangleleft u]$, we define $x(\triangleleft) = 1$ if the term is an ES, and $x(\triangleleft) = 0$ otherwise.

We use two notions of **contexts**. Term contexts $\mathtt{C}$ extend those of the $\lambda$-calculus to explicit cuts. List contexts $\mathtt{L}$ denote an arbitrary list of explicit cuts. They will be used to implement reduction *at a distance* in the operational semantics defined ahead.

**Free/bound variables** of terms are defined as usual, notably $\mathtt{fv}(t[x\triangleleft u]) := \mathtt{fv}(t)\backslash\{x\} \cup \mathtt{fv}(u)$. These notions are extended to contexts as expected, in particular $\mathtt{fv}(\Box) := \emptyset$. The **domain** of a **list context** is given by $\mathtt{dlc}(\Box) := \emptyset$ and $\mathtt{dlc}(\mathtt{L}[x \triangleleft u]) := \mathtt{dlc}(\mathtt{L}) \cup \{x\}$. $\alpha$-conversion [13] is extended to $\lambda\mathtt{R}$-terms as expected and used to avoid capture of free variables. We write $t\{x\backslash u\}$ for the meta-level (capture-free) substitution simultaneously replacing all the free occurrences of the variable $x$ in $t$ by the term $u$.

The **application of a context** $\mathtt{C}$ **to a term** $t$, written $\mathtt{C}\langle t\rangle$, replaces the hole $\Box$ of $\mathtt{C}$ by $t$. For instance, $\Box\langle t\rangle = t$ and $(\lambda x.\Box)\langle t\rangle = \lambda x.t$. This operation is not defined modulo $\alpha$-conversion, so that capture of variables eventually happens. Thus, we also consider another kind of application of contexts to terms, denoted with double brackets, which is only defined if there is no capture of variables. For instance, $(\lambda y.\Box)\langle\!\langle x\rangle\!\rangle = \lambda y.x$ while $(\lambda x.\Box)\langle\!\langle x\rangle\!\rangle$ is undefined.

**Operational semantics.** ES may block some expected *meaningful* (*i.e.* non-structural) reductions. For instance, $\beta$-reduction is blocked in $(\lambda x.t)[y\backslash v]u$ because an ES lies between the function and its argument. This kind of stuck redexes do not happen in graphical representations (*e.g.* [28]), but it is typical in the sequential structure of *term* syntaxes.

There are at least two ways to handle this issue. The first one is based on *structural/permutation* rules, as in [33], where the substitution is first pushed outside the application node, as $(\lambda x.t)[y\backslash v]u \to ((\lambda x.t)u)[y\backslash v]$, so that $\beta$-reduction is finally unblocked. The second, less elementary, possibility is given by an operational semantics *at a distance* [6,4], where the $\beta$-rule can be fired by a rule like $\mathtt{L}\langle\lambda x.t\rangle u \to \mathtt{L}\langle t[x\backslash u]\rangle$, $\mathtt{L}$ being an arbitrary list context. The distance paradigm is therefore used to gather meaningful and permutation rules in only one reduction step. In $\lambda\mathtt{R}$, we combine these two technical tools. First, we consider the following permutation rules, all of them are constrained by the condition $x \notin \mathtt{fv}(t)$.

$$\begin{array}{llllll} \lambda x.u[y \triangleleft t] & \mapsto_\pi & (\lambda x.u)[y \triangleleft t] & v[x \triangleleft u]t & \mapsto_\pi & (vt)[x \triangleleft u] \\ tv[x \triangleleft u] & \mapsto_\pi & (tv)[x \triangleleft u] & t[y \triangleleft v[x \triangleleft u]] & \mapsto_\pi & t[y \triangleleft v][x \triangleleft u] \end{array}$$

The reduction relation $\to_\pi$ is defined as the closure of the rules $\mapsto_\pi$ under *all* contexts. It does not hold any computational content, only a structural one that unblocks redexes by moving explicit cuts out.

In order to highlight the computational content of node replication we combine distance and permutations within the $\lambda\mathtt{R}$**-calculus**, given by the closure of the following rules by all the contexts.

$$\begin{array}{llll} \mathtt{L}\langle\lambda x.t\rangle u & \mapsto_{\mathtt{dB}} & \mathtt{L}\langle t[x\backslash u]\rangle & \\ t[x\backslash \mathtt{L}\langle uv\rangle] & \mapsto_{\mathtt{app}} & \mathtt{L}\langle t\{x\backslash yz\}[y\backslash u][z\backslash v]\rangle & \text{where } y \text{ and } z \text{ are fresh} \\ t[x\backslash \mathtt{L}\langle \lambda y.u\rangle] & \mapsto_{\mathtt{dist}} & \mathtt{L}\langle t[x\backslash\!\backslash \lambda y.z[z\backslash u]]\rangle & \text{where } z \text{ is fresh} \\ t[x\backslash\!\backslash \lambda y.u] & \mapsto_{\mathtt{abs}} & \mathtt{L}\langle t\{x\backslash \lambda y.p\}\rangle & \text{where } u \to_\pi^* \mathtt{L}\langle p\rangle \text{ and } y \notin \mathtt{fv}(\mathtt{L}) \\ t[x\backslash \mathtt{L}\langle y\rangle] & \mapsto_{\mathtt{var}} & \mathtt{L}\langle t\{x\backslash y\}\rangle & \end{array}$$

Notice in the five rules above that the (meta-level) substitution is *full* (it is performed simultaneously on all free occurrences of the variable $x$), and the list context $\mathtt{L}$ is always pushed outside the term $t$. We will highlight in *green* such list contexts in the forthcoming examples to improve readability. Apart from rule $\mathtt{dB}$ used to fire $\beta$-reductions, there are four substitution rules used to copy abstractions, applications and variables, pushing outside all the cuts surrounding the node to be copied. Rule $\mathtt{app}$ copies one application node, while rule $\mathtt{var}$ copies one variable node. The case of abstractions is more involved as explained below.

The specificity in copying an abstraction $\lambda y.u$ is due to the (binding) relation between $\lambda y$ and all the free occurrences of $y$ in its body $u$. Abstractions are thus copied in two stages. The first one is implemented by the rule `dist`, creating a distributor in which a potentially replaceable abstraction is placed, while moving its body inside a new ES. There are then two ways to replicate nodes of the body. Either they can be copied inside the distributor (where the binding relation between $\lambda y$ and the bound occurrences of $y$ is kept intact), or they can be pushed outside the distributor, by means of the (non-deterministic) rule `abs`. In the second case, however, free occurrences of $y$ cannot be pushed outside the abstraction (with binder $y$) to be duplicated, at the risk of breaking consistency: only shared components without $y$ links can be then pushed outside. These components are gathered together into a list context `L`, which is pushed outside by using permutation rules, before performing the substitution of the pure body containing all the bound occurrences of $y$. Specifying this operation using only distance is hard, thus permutation rules are also used in our rule `abs`.

The `s`-substitution relation $\to_{\mathtt{s}}$ (resp. distant Beta relation $\to_{\mathtt{dB}}$) is defined as the closure of $\mapsto_{\mathtt{app}} \cup \mapsto_{\mathtt{dist}} \cup \mapsto_{\mathtt{abs}} \cup \mapsto_{\mathtt{var}}$ (resp. $\mapsto_{\mathtt{dB}}$) under *all* contexts, and the reduction relation $\to_{\mathtt{R}}$ is the union of $\to_{\mathtt{s}}$ and $\to_{\mathtt{dB}}$.

*Example 1.* Let $t_0 = (\lambda x_1.x_1)(\lambda y.\mathtt{I}y)$. In what follows, we underline the term where the reduction is performed:

$$t_0 \to_{\mathtt{dB}} \underline{x_1[x_1\backslash\lambda y.\mathtt{I}y]} \to_{\mathtt{dist}} x_1[x_1\backslash\!\!\backslash\lambda y.\underline{z[z\backslash\mathtt{I}y]}] \to_{\mathtt{app}} x_1[x_1\backslash\!\!\backslash\lambda y.\underline{(z_1z_2)[z_1\backslash\mathtt{I}]}[z_2\backslash y]]$$
$$\to_{\mathtt{dist}} x_1[x_1\backslash\!\!\backslash\lambda y.\underline{(z_1z_2)[z_1\backslash\!\!\backslash\lambda x_3.z_3[z_3\backslash x_3]][z_2\backslash y]}]$$
$$\to_{\mathtt{var}} \underline{x_1[x_1\backslash\!\!\backslash\lambda y.(z_1y)[z_1\backslash\!\!\backslash\lambda x_3.z_3[z_3\backslash x_3]]]} \to_{\mathtt{abs}} (\lambda y.z_1y)[z_1\backslash\!\!\backslash\lambda x_3.z_3[z_3\backslash x_3]]$$

Let $\mathcal{R}$ be any reduction relation. We write $\to_{\mathcal{R}}^*$ for the reflexive-transitive closure of $\to_{\mathcal{R}}$. A term $t$ is said to be **$\mathcal{R}$-confluent** *iff* $t \to_{\mathcal{R}}^* u$ and $t \to_{\mathcal{R}}^* s$ implies there is $t'$ such that $u \to_{\mathcal{R}}^* t'$ and $s \to_{\mathcal{R}}^* t'$. The relation $\mathcal{R}$ is **confluent** *iff* every term is $\mathcal{R}$-confluent. A term $t$ is said to be in **$\mathcal{R}$-normal form** (written also $\mathcal{R}$-nf) *iff* there is no $t'$ such that $t \to_{\mathcal{R}} t'$. A term $t$ is said to be **$\mathcal{R}$-terminating** or **$\mathcal{R}$-normalizing** *iff* there is no infinite $\mathcal{R}$-sequence starting at $t$. The reduction $\mathcal{R}$ is said to be **terminating** *iff* every term is $\mathcal{R}$-terminating.

**Levels.** The notion of level plays a key role in this work. Intuitively, the level of a variable in a term indicates the maximal depth of its free occurrences w.r.t. ES (and not w.r.t. explicit distributors). However, in order to keep soundness w.r.t. the permutation rules, levels are computed along *linked chains* of ES. For instance, the level of $w$ in both $x[x\backslash y[y\backslash w]]$ and $x[x\backslash y][y\backslash w]$ is 2. Formally, the **level** of a variable $z$ in a term $t$ is defined by (structural) induction, while assuming by $\alpha$-conversion that $z$ is not a bound variable in $t$:

$$\mathtt{lv}_z(x) := 0 \qquad \mathtt{lv}_z(t_1t_2) := \max(\mathtt{lv}_z(t_1), \mathtt{lv}_z(t_2)) \qquad \mathtt{lv}_z(\lambda y.t) := \mathtt{lv}_z(t)$$
$$\mathtt{lv}_z(t[x \lhd u]) := \begin{cases} \mathtt{lv}_z(t) & \text{if } z \notin \mathtt{fv}(u) \\ \max(\mathtt{lv}_z(t), \mathtt{lv}_x(t) + \mathtt{lv}_z(u) + x(\lhd)) & \text{otherwise} \end{cases}$$

Notice that $\mathtt{lv}_w(t) = 0$ whenever $w \notin \mathtt{fv}(t)$ or $t$ is pure. We illustrate the concept of level by an example. Consider $t = x[x\backslash z[y\backslash w]][w\backslash w']$, then $\mathtt{lv}_z(t) = 1$, $\mathtt{lv}_{w'}(t) = 3$ and $\mathtt{lv}_y(t) = 0$ because $y \notin \mathtt{fv}(t)$. This notion is also extended to contexts as expected, *i.e.* $\mathtt{lv}_\Box(\mathtt{C}) = \mathtt{lv}_z(\mathtt{C}\langle\langle z\rangle\rangle)$, where $z$ is a fresh variable.

**Lemma 2.** *Let $t \in \Lambda_{\mathtt{R}}$. If $t_0 \rightarrow_{\pi,\mathtt{s}} t_1$, then $\mathtt{lv}_w(t_0) \geq \mathtt{lv}_w(t_1)$ for any $w \in \mathcal{X}$.*

It is worth noticing that there are two cases when the level of a variable in a term may decrease: using a permutation rule to push an explicit cut out of another cut when the first one is a void cut, or using rule $\mapsto_{\mathtt{var}}$.

Hence, levels alone are not enough to prove termination of $\rightarrow_{\mathtt{s}}$. We then define a decreasing measure for $\rightarrow_{\mathtt{s}}$ in which not only variables are indexed by a level, but also constructors. For instance, in $t[x\backslash \lambda y.yz]$, we can consider that the level of *all* the constructors of $\lambda y.yz$ have level $\mathtt{lv}_x(t)$. This will ensure that the level of an abstraction will decrease when applying rule dist, as well as the level of an application when applying rule app. This is what we do next.

## 3 Operational Properties

We now prove three key properties of the $\lambda\mathtt{R}$-calculus: termination of the reduction system $\rightarrow_{\mathtt{s}}$, relation between $\lambda\mathtt{R}$ and the $\lambda$-calculus, and confluence of the reduction system $\rightarrow_{\lambda\mathtt{R}}$.

**Termination of $\rightarrow_{\mathtt{s}}$.** Some (rather informal) arguments are provided in [33] to justify termination of the substitution subrelation of their whole calculus. We expand these ideas into an alternative full formal proof adapted to our case, which is based on a measure being strictly decreasing w.r.t. $\rightarrow_{\mathtt{s}}$.

We consider a set $\mathcal{O}$ of objects of the form $\mathtt{a}(k, n)$ or $\mathtt{b}(k)$ ($k, n \in \mathbb{N}$), which is equipped with the following ordering $>^{\mathcal{O}}$:

$$\begin{array}{llll} \mathtt{a}(k,n) >^{\mathcal{O}} \mathtt{a}(k',n) & \text{if } k > k', \text{ or } (k = k' \text{ and } n > n') & \mathtt{b}(k) >^{\mathcal{O}} \mathtt{a}(k',n) & \text{if } k \geq k' \\ \mathtt{a}(k,n) >^{\mathcal{O}} \mathtt{b}(k') & \text{if } k > k' & \mathtt{b}(k) >^{\mathcal{O}} \mathtt{b}(k') & \text{if } k > k' \end{array}$$

**Lemma 3.** *The order $>^{\mathcal{O}}$ on the set $\mathcal{O}$ is well-founded.*

We write $>^{\mathcal{O}}_{\mathtt{MUL}}$ for the multiset extension of the order $>^{\mathcal{O}}$ on $\mathcal{O}$, which turns out to be well-founded [8] by Lem. 3. We are now ready to (inductively) define our **cuts level** measure $\mathsf{C}\,(\_)$ on terms, where the following operation on multisets is used $p \cdot M := [\mathtt{a}(p+k, n) \mid \mathtt{a}(k, n) \in M] \sqcup [\mathtt{b}(p+k) \mid \mathtt{b}(k) \in M]$, where $\sqcup$ denotes multiset union.

$$\begin{array}{l} \mathsf{C}\,(x) := [\,] \qquad \mathsf{C}\,(\lambda x.t) := \mathsf{C}\,(t) \qquad \mathsf{C}\,(tu) := \mathsf{C}\,(t) \sqcup \mathsf{C}\,(u) \\ \mathsf{C}\,(t[x\backslash u]) := \mathsf{C}\,(t) \sqcup (\mathtt{lv}_x(t)+1) \cdot \mathsf{C}\,(u) \sqcup [\mathtt{a}(\mathtt{lv}_x(t)+1, |u|)] \\ \mathsf{C}\,(t[x\backslash\backslash u]) := \mathsf{C}\,(t) \sqcup \mathtt{lv}_x(t) \cdot \mathsf{C}\,(u) \sqcup [\mathtt{b}(\mathtt{lv}_x(t))] \end{array}$$

Intuitively, the integer $k$ in $\mathtt{a}(k, n)$ and $\mathtt{b}(k)$ counts the level of variables bound by explicit cuts, while $n$ counts the size of terms to be substituted by an ES. Remark that for every pure term $p$ we have $\mathsf{C}\,(p) = [\,]$. Moreover:

**Lemma 4.** *Let $t_0 \in \Lambda_{\mathtt{R}}$. Then $t_0 \to_\pi t_1$ (resp. $t_0 \to_{\mathtt{s}} t_1$) implies $\mathsf{C}(t_0) \geq^{\mathcal{O}}_{\mathtt{MUL}} \mathsf{C}(t_1)$ (resp. $\mathsf{C}(t_0) >^{\mathcal{O}}_{\mathtt{MUL}} \mathsf{C}(t_1)$).*

As an example, consider the following reduction sequence:

$$\begin{array}{llll} t_0 = (yy)[y\backslash(\lambda z.x)w] & \to_{\mathtt{app}} & (y_1y_2)(y_1y_2)[y_1\backslash\lambda z.x][y_2\backslash w] = t_1 \to_{\mathtt{var}} \\ t_2 = (y_1w)(y_1w)[y_1\backslash\lambda z.x] & \to_{\mathtt{dist}} & (y_1w)(y_1w)[y_1\backslash\!\!\backslash\lambda z.r[r\backslash x]] \quad = t_3 \end{array}$$

We have $\mathsf{C}(t_0) = [\mathtt{a}(1,4)]$, $\mathsf{C}(t_1) = [\mathtt{a}(1,1), \mathtt{a}(1,2)]$, $\mathsf{C}(t_2) = [\mathtt{a}(1,2)]$, $\mathsf{C}(t_3) = [\mathtt{a}(1,1), \mathtt{b}(0)]$. So $\mathsf{C}(t_i) >_{\mathtt{MUL}} \mathsf{C}(t_{i+1})$ for $i = 0, 1, 2, 3$.

**Corollary 5.** *The reduction relation $\to_{\mathtt{s}}$ is terminating.*

**Simulations.** We show the relation between $\lambda\mathtt{R}$ and the $\lambda$-calculus, as well as the atomic $\lambda$-calculus. For that, we introduce a projection from $\lambda\mathtt{R}$-terms to $\lambda$-terms implementing the unfolding of all the explicit cuts: $x^\downarrow := x$, $(\lambda x.t)^\downarrow := \lambda x.t^\downarrow$, $(tu)^\downarrow := t^\downarrow u^\downarrow$, $(t[x \triangleleft u])^\downarrow := t^\downarrow\{x\backslash u^\downarrow\}$. Thus *e.g.* $x[x\backslash z][y\backslash w][w\backslash w']^\downarrow = z$.

**Lemma 6.** *Let $t_0 \in \Lambda_{\mathtt{R}}$. If $t_0 \to_{\mathtt{R}} t_1$, then $t_0^\downarrow \to_\beta^* t_1^\downarrow$. In particular, if either $t_0 \to_\pi t_1$ or $t_0 \to_{\mathtt{s}} t_1$, then $t_0^\downarrow = t_1^\downarrow$.*

The relation $\to_{\mathtt{s}}$ enjoys **full composition** on *pure* terms, namely, for any $p \in \Lambda$, $t[x\backslash p] \to_{\mathtt{s}}^+ t\{x\backslash p\}$. This property does not hold in general. Indeed, if $t = xx$, then $(xx)[x\backslash z[z\backslash w]]$ does not $\mathtt{s}$-reduce to $(z[z\backslash w])(z[z\backslash w])$, but to $(zz)[z\backslash w]$. However, full composition restricted to pure terms is sufficient to prove simulation of the $\lambda$-calculus.

**Lemma 7 (Simulation of the $\lambda$-calculus).** *Let $p_0 \in \Lambda$. If $p_0 \to_\beta p_1$, then $p_0 \to_{\mathtt{dB}}\to_{\mathtt{s}}^+ p_1$.*

The previous results have an important consequence relating the original atomic $\lambda$-calculus and the $\lambda\mathtt{R}$-calculus. Indeed, it can be shown that reduction in the atomic $\lambda$-calculus is captured by $\lambda\mathtt{R}$, and vice-versa. More precisely, the $\lambda\mathtt{R}$-calculus can be simulated into the atomic $\lambda$-calculus by Lem. 6 and [33], while the converse holds by [33] and Lem. 7.

A more structural correspondence between $\lambda\mathtt{R}$ and the atomic $\lambda$-calculus could also be established. Indeed, $\lambda\mathtt{R}$ can be first refined into a (non-linear) calculus *without* distance, let say $\lambda\mathtt{R}'$, so that permutation rules are integrated in the intermediate calculus as independent rules. Then a structural relation can be established between $\lambda\mathtt{R}$ and $\lambda\mathtt{R}'$ on one side, and $\lambda\mathtt{R}'$ and the atomic $\lambda$-calculus on the other side (as for example done in [43] for the $\lambda$-calculus).

**Confluence.** By Cor. 5 the reduction relation $\to_{\mathtt{s}}$ is terminating. It is then not difficult to conclude confluence of $\to_{\mathtt{s}}$ by using the unfolding function $\_^\downarrow$. Therefore, by termination of $\to_{\mathtt{s}}$ any $t \in \Lambda_{\mathtt{R}}$ has an $\mathtt{s}$-nf, and by confluence this $\mathtt{s}$-nf is unique (and computed by the unfolding function). Using the interpretation method [35] together with Lem. 6, Cor. 5, and Lem. 7, one obtains:

**Theorem 8.** *The reduction relation $\to_{\mathtt{R}}$ is confluent.*

## 4 Encoding Evaluation Strategies

In the theory of programming languages [56], the notion of *calculus* is usually based on a non-deterministic rewriting relation, providing an equational system of calculation, while the deterministic notion of *strategy* is associated to a concrete machinery being able to implement a specific evaluation procedure. Typical evaluation strategies are call-by-name, call-by-value, call-by-need, etc.

Although the atomic $\lambda$-calculus was introduced as a technical tool to implement full laziness, only its (non-deterministic) equational theories was studied. In this paper we bridge the gap between the theoretical presentation of the atomic $\lambda$-calculus and concrete specifications of evaluation strategies. Indeed, we use the $\lambda\mathtt{R}$-calculus to investigate two concrete cases: a call-by-name strategy implementing weak head reduction, based on full substitution, and the call-by-need fully lazy strategy, which uses linear substitution.

In both cases, explicit cuts can in principle be placed anywhere in the distributors, thus demanding to dive deep in such terms to deal with them. We then restrict the set of terms to a subset $\mathtt{U}$, which simplifies the formal reasoning of explicit cuts inside distributors. Indeed, distributors will all be of the shape $[x\backslash\!\!\backslash \lambda y.\mathtt{L}\langle p\rangle]$, where $p$ is a pure term (and $\mathtt{L}$ is a *commutative list* defined below). We argue that this restriction is natural in a weak implementation of the $\lambda$-calculus: it is true on pure terms and is preserved through evaluation. We consider the following grammars.

$$\begin{array}{lll} \textbf{(Linear Cut Values)} & \mathtt{T} & ::= \lambda x.\mathtt{LL}\langle p\rangle \text{ where } y \in \mathtt{dlc}(\mathtt{LL}) \implies |p|_y = 1 \\ \textbf{(Commutative Lists)} & \mathtt{LL} & ::= \Box \mid \mathtt{LL}[x\backslash p] \mid \mathtt{LL}[x\backslash\!\!\backslash \mathtt{T}] \text{ where } |\mathtt{LL}|_x = 0 \\ \textbf{(Values)} & v & ::= \lambda x.p \\ \textbf{(Restricted Terms)} & \mathtt{U} & ::= x \mid v \mid \mathtt{UU} \mid \mathtt{U}[x\backslash \mathtt{U}] \mid \mathtt{U}[x\backslash\!\!\backslash \mathtt{T}] \end{array}$$

A term $t$ generated by any of the grammars $G$ defined above is written $t \in G$. Thus *e.g.* $\lambda x.(yz)[y\backslash \mathtt{I}][z\backslash \mathtt{I}] \in \mathtt{T}$ but $\lambda x.(yy)[y\backslash \mathtt{I}] \notin \mathtt{T}$, $\Box[x\backslash yz][x'\backslash \mathtt{I}] \in \mathtt{LL}$ but $\Box[x\backslash yz][y\backslash \mathtt{I}] \notin \mathtt{LL}$, and $(yz)[y\backslash\!\!\backslash \mathtt{I}] \in \mathtt{U}$ but $(yz)[y\backslash\!\!\backslash \lambda x.(yy)[y\backslash \mathtt{I}]] \notin \mathtt{U}$.

The set $\mathtt{T}$ is stable by the relation $\to_{\mathtt{s}}$, but $\mathtt{U}$ is clearly not stable under the whole $\to_{\mathtt{R}}$ relation, where $\mathtt{dB}$-reductions may occur under abstractions. However, $\mathtt{U}$ is stable under both weak strategies to be defined: call-by-name and call-by-need. We factorize the proofs by proving stability for a more general relation $\to_{\mathtt{R}'}$, defined as the relation $\to_{\mathtt{R}}$ with $\mathtt{dB}$-reductions forbidden under abstractions and inside distributors.

**Lemma 9 (Stability of the Grammar by $\to_{\mathtt{s}}/\to_{\mathtt{R}'}$).**

1. *If $t \in \mathtt{T}$ and $t \to_{\mathtt{s}} t'$, then $t' \in \mathtt{T}$.*
2. *If $t \in \mathtt{U}$ and $t \to_{\mathtt{R}'} t'$, then $t' \in \mathtt{U}$.*

### 4.1 Call-by-name

The **call-by-name** (CBN) strategy $\to_{\mathtt{name}}$ (Fig. 1) is defined on the set of terms $\mathtt{U}$ as the union of the following relations $\to_{\mathtt{ndb}}$ and $\to_{\mathtt{ns}}$. The strategy is *weak* as there is no reduction under abstractions. It is also worth noticing (as a particular case of Lem. 9) that $t \in \mathtt{U}$ and $t \to_{\mathtt{name}} t'$ implies $t' \in \mathtt{U}$.

$$\frac{t \mapsto_{\mathtt{dB}} t'}{t \to_{\mathtt{ndb}} t'}\ (\mathtt{dB}) \qquad \frac{t \to_{\mathtt{ndb}} t'}{tu \to_{\mathtt{ndb}} t'u}\ (\mathtt{app\_dB}) \qquad \frac{t \to_{\mathtt{ndb}} t'}{t[x \triangleleft u] \to_{\mathtt{ndb}} t'[x \triangleleft u]}\ (\mathtt{sub\_dB})$$

$$\frac{t \mapsto_{\mathtt{s}} t'}{t \to_{\mathtt{ns}} t'}\ (\mathtt{s}) \qquad \frac{t \to_{\mathtt{ns}} t'}{tu \to_{\mathtt{ns}} t'u}\ (\mathtt{app\_s}) \qquad \frac{t \to_{\mathtt{ns}} t'}{u[x \backslash\!\backslash \lambda y.t] \to_{\mathtt{ns}} u[x \backslash\!\backslash \lambda y.t']}\ (\mathtt{sub\_s})$$

**Fig. 1.** Call-by-Name Strategy

*Example 10.* Let $t_0 = (\lambda x_1.\mathtt{I}(x_1\mathtt{I}))(\lambda y.\mathtt{I}y)$. Then,

$$t_0 \to_{\mathtt{dB}}^{(\mathtt{dB})} \underline{(\mathtt{I}(x_1\mathtt{I}))[x_1\backslash\lambda y.\mathtt{I}y]} \to_{\mathtt{dist}}^{(\mathtt{s})} (\mathtt{I}(x_1\mathtt{I}))[x_1\backslash\!\backslash\lambda y.\underline{z[z\backslash\mathtt{I}y]}] \to_{\mathtt{app}}^{(\mathtt{sub\_s})}$$
$$(\mathtt{I}(x_1\mathtt{I}))[x_1\backslash\!\backslash\lambda y.\underline{(z_1z_2)[z_1\backslash\mathtt{I}]}[z_2\backslash y]] \to_{\mathtt{var}}^{(\mathtt{sub\_s})} \underline{(\mathtt{I}(x_1\mathtt{I}))[x_1\backslash\!\backslash\lambda y.(z_1y)\ [z_1\backslash\mathtt{I}]\ ]} \to_{\mathtt{abs}}^{(\mathtt{s})}$$
$$(\underline{\mathtt{I}((\lambda y.z_1y)\mathtt{I})})[z_1\backslash\mathtt{I}] \to_{\mathtt{dB}}^{(\mathtt{sub\_dB})} x_2[x_2\backslash(\lambda y.z_1y)\mathtt{I}][z_1\backslash\mathtt{I}]$$

Although the strategy $\to_{\mathtt{name}}$ is not deterministic, it enjoys the remarkable *diamond* property, guaranteeing in particular that all reduction sequences starting from $t$ and ending in a normal form have the same length.

It is worth noticing that simulation lemmas also hold between call-by-name in the $\lambda$-calculus, known as weak head reduction and denoted by $\to_{\mathtt{whr}}$, and the $\lambda\mathtt{R}$-calculus. Indeed, $\to_{\mathtt{whr}}$ is defined as the $\beta$-reduction rule closed by contexts $\mathtt{E} ::= \Box \mid \mathtt{E}\,t$. Then, as a consequence of Lem. 7, we have that $p_0 \to_{\mathtt{whr}} p_1$ implies $p_0 \to_{\mathtt{R}}^* p_1$, and as a consequence of Lem. 6, we have that $t_0 \to_{\mathtt{name}} t_1$ implies $t_0^{\downarrow} \to_{\beta}^* t_1^{\downarrow}$. More importantly, call-by-name in the $\lambda$-calculus and call-by-name in the $\lambda\mathtt{R}$-calculus are also related. Indeed,

**Lemma 11 (Relating Call-by-Name Strategies).**

– *Let* $p_0 \in \Lambda$. *If* $p_0 \to_{\mathtt{whr}} p_1$ *then* $p_0 \to_{\mathtt{name}}^+ p_1$.
– *Let* $t_0 \in \mathcal{U}$. *If* $t_0 \to_{\mathtt{name}} t_1$ *then* $t_0^{\downarrow} \to_{\mathtt{whr}}^* t_1^{\downarrow}$.

## 4.2 Call-by-need

We now specify a deterministic strategy `flneed` implementing demand-driven computations and only linearly replicating nodes of *values* (*i.e.* pure abstractions). Given a value $\lambda x.p$, only the piece of structure containing the paths between the binder $\lambda x$ and all the free occurrences of $x$ in $p$, named *skeleton*, will be copied. All the other components of the abstraction will remain shared, thus avoiding some future duplications of redexes, as explained in the introduction. By copying only the smallest possible substructure of the abstraction, the strategy `flneed` implements an optimization of call-by-need called `f`*ully* `l`*azy sharing* [60]. First, we formally define the key notions we are going to use.

A **free expression** [39,9] of a *pure* term $p$ is a strict subterm $q$ of $p$ such that every free occurrence of a variable in $q$ is also a free occurrence of the variable in $p$. A **free expression** of $p$ is **maximal** if it is not a subterm of another free expression of $p$. From now on, we will consider the multiset of all maximal free expressions (**MFE**) of a term. Thus *e.g.* the MFEs of $\lambda y.p$, where $p = (\mathtt{I}y)\mathtt{I}(\lambda z.zyw)$, is given by the multiset $[\mathtt{I}, \mathtt{I}, w]$.

An **$n$-ary context** ($n \geq 0$) is a term with $n$ holes $\Box$. A skeleton is an $n$-ary pure context where the maximal free expressions w.r.t. a variable set $\theta$ are replaced with holes. Formally, the **$\theta$-skeleton** $\{\!\{p\}\!\}^\theta$ of a pure term $p$, where $\theta = \{x_1 \dots x_n\}$, is the n-ary pure context $\{\!\{p\}\!\}^\theta$ such that $\{\!\{p\}\!\}^\theta\langle q_1, \dots, q_n\rangle = p$, for $[q_1, \dots, q_n]$ the maximal free expressions of $\lambda x_1. \dots \lambda x_n.p$ [4]. Thus, for the same $p$ as before, $\lambda y.\{\!\{p\}\!\}^y = \lambda y.(\Box y)\Box(\lambda z.zy\Box)$.

**The Splitting Operation.** Splitting a term into a skeleton and a multiset of MFEs is at the core of full laziness. This can naturally be implemented in the node replication model, as observed in [33]. Here, we define a (small-step) strategy $\to_{\mathtt{st}}$ on the set of terms $\mathtt{T}$ to achieve it (Fig. 2), which is indeed a subset of the reduction relation $\lambda\mathtt{R}$[5]. The relation $\to_{\mathtt{st}}$ makes use of four basic rules which are parameterized by the variable $y$ upon which the skeleton is built, written $\mapsto^y$. There are also two contextual (inductive) rules.

$$\frac{}{t[x\backslash y] \mapsto^y_{\mathtt{var}} t\{x\backslash y\}} \qquad \frac{y \in \mathtt{fv}(p_1p_2)}{t[x\backslash p_1p_2] \mapsto^y_{\mathtt{app}} t\{x\backslash x_1x_2\}[x_1\backslash p_1][x_2\backslash p_2]}$$

$$\frac{y \in \mathtt{fv}(\lambda z.p)}{t[x\backslash \lambda z.p] \mapsto^y_{\mathtt{dist}} t[x\backslash\!\!\backslash \lambda z.w[w\backslash p]]} \qquad \frac{y \in \mathtt{fv}(\lambda z.\mathtt{LL}\langle p\rangle) \quad z \notin \mathtt{fv}(\mathtt{LL})}{t[x\backslash\!\!\backslash \lambda z.\mathtt{LL}\langle p\rangle] \mapsto^y_{\mathtt{abs}} \mathtt{LL}\langle t\{x\backslash \lambda z.p\}\rangle}$$

$$\frac{t \mapsto^y t' \quad y \in \mathtt{fv}(t) \quad y \notin \mathtt{fv}(\mathtt{LL})}{\lambda y.\mathtt{LL}\langle t\rangle \to_{\mathtt{st}} \lambda y.\mathtt{LL}\langle t'\rangle}\,\mathtt{ctx}_1 \qquad \frac{t \to_{\mathtt{st}} t' \quad y \in \mathtt{fv}(t) \quad y \notin \mathtt{fv}(\mathtt{LL})}{\lambda y.\mathtt{LL}\langle u[x\backslash\!\!\backslash t]\rangle \to_{\mathtt{st}} \lambda y.\mathtt{LL}\langle u[x\backslash\!\!\backslash t']\rangle}\,\mathtt{ctx}_2$$

**Fig. 2.** Relation $\to_{\mathtt{st}}$: Splitting Skeleton and MFEs in Small-Step Semantics

*Example 12.* Let $y, z \notin \mathtt{fv}(t)$, so that $t$ is the MFE of $\lambda y.x[x\backslash \lambda z.(yt)z]$. Then,

$$\begin{array}{l}
\lambda y.\underline{x[x\backslash \lambda z.(yt)z]} \to^y_{\mathtt{dist}} \lambda y.x[x\backslash\!\!\backslash \lambda z.\underline{w[w\backslash (yt)z]}] \to^z_{\mathtt{app}} \\
\lambda y.x[x\backslash\!\!\backslash \lambda z.\underline{(w_1w_2)[w_1\backslash yt][w_2\backslash z]}] \to^z_{\mathtt{var}} \lambda y.\overline{x[x\backslash\!\!\backslash \lambda z.(w_1z)\,[w_1\backslash yt]\,]} \to^y_{\mathtt{abs}} \\
\lambda y.\underline{(\lambda z.w_1z)[w_1\backslash yt]} \to^y_{\mathtt{app}} \lambda y.\underline{(\lambda z.(x_1x_2)z)[x_1\backslash y]}[x_2\backslash t] \to^y_{\mathtt{var}} \lambda y.(\lambda z.(yx_2)z)[x_2\backslash t]
\end{array}$$

Notice that the focused variable changes from $y$ to $z$, then back to $y$. This is because $\to_{\mathtt{st}}$ constructs the innermost skeletons first.

**Lemma 13.** *The reduction relation $\to_{\mathtt{st}}$ is confluent and terminating.*

Thus, from now on, we denote by $\Downarrow_{\mathtt{st}}$ the function relating a term of $\mathtt{T}$ to its unique $\mathtt{st}$-nf.

[4] The order of variables in the set $\theta$ is indeed irrelevant.

[5] Since $\to_{\mathtt{st}}$ acts only on terms in $\mathtt{T}$, it is handled by linear substitution.

**Lemma 14 (Correctness of $\rightarrow_{\mathtt{st}}$).** *Let $p \in \Lambda$ and $q_1, \dots, q_n$ be the MFEs of $\lambda y.p$. Then $\lambda y.z[z\backslash p] \Downarrow_{\mathtt{st}} \lambda y.\{\!\{p\}\!\}^{\{y\}}\langle x_1, \dots, x_n\rangle[x_i\backslash q_i]_{i \leq n}$ where the variables $x_1, \dots, x_n$ are fresh and pairwise distinct.*

Since the small-step semantics is contained in the $\lambda\mathtt{R}$-calculus, we use it to build our call-by-need strategy of $\lambda\mathtt{R}$.

**The strategy.** The **call-by-need strategy** $\rightarrow_{\mathtt{flneed}}$ (Fig. 3) is defined on the set of terms $\mathtt{U}$, by using closure under the *need contexts*, given by the grammar $\mathtt{N} ::= \Box \mid \mathtt{N}t \mid \mathtt{N}[x \triangleleft t] \mid \mathtt{N}\langle\!\langle x\rangle\!\rangle[x\backslash\mathtt{N}]$, where $\mathtt{N}\langle\!\langle \_\rangle\!\rangle$ denotes capture-free application of contexts (Sec. 2). As for call-by-name (Sec. 4.1), the call-by-need strategy is *weak*, because no *meaningful* reduction steps are performed under abstractions.

$$
\begin{array}{lll}
\mathtt{L}\langle\lambda x.p\rangle u & \mapsto_{\mathtt{dB}} & \mathtt{L}\langle p[x\backslash u]\rangle \\
\mathtt{N}\langle\!\langle x\rangle\!\rangle[x\backslash\mathtt{L}\langle\lambda y.p\rangle] & \mapsto_{\mathtt{spl}} & \mathtt{L}\langle\mathtt{LL}\langle\mathtt{N}\langle\!\langle x\rangle\!\rangle[x\backslash\!\!\backslash\lambda y.p']\rangle\rangle \quad \text{if } \lambda y.z[z\backslash p] \Downarrow_{\mathtt{st}} \lambda y.\mathtt{LL}\langle p'\rangle \\
\mathtt{N}\langle\!\langle x\rangle\!\rangle[x\backslash\!\!\backslash v] & \mapsto_{\mathtt{sub}} & \mathtt{N}\langle\!\langle v\rangle\!\rangle[x\backslash\!\!\backslash v]
\end{array}
$$

**Fig. 3.** Call-by-Need Strategy

Rule $\mathtt{dB}$ is the same one used to define $\mathtt{name}$. Although rules $\mathtt{spl}$ and $\mathtt{sub}$ could have been presented in a unique rule of the form $\mathtt{N}\langle\!\langle x\rangle\!\rangle[x\backslash\mathtt{L}\langle\lambda y.p\rangle] \mapsto \mathtt{L}\langle\mathtt{LL}\langle\mathtt{N}\langle\!\langle\lambda y.p'\rangle\!\rangle[x\backslash\!\!\backslash\lambda y.p']\rangle\rangle$, we prefer to keep them separate since they represent different stages in the strategy. Indeed, rule $\mathtt{spl}$ only uses node replication operations to compute the skeleton of the abstraction, while rule $\mathtt{sub}$ implements one-shot *linear* substitution.

Notice that as a particular case of Lem. 9, $t \in \mathtt{U}$ and $t \rightarrow_{\mathtt{flneed}} t'$ implies $t' \in \mathtt{U}$. Another interesting property is that $t \rightarrow_{\mathtt{sub}} t'$ implies $\mathtt{lv}_z(t) \geq \mathtt{lv}_z(t')$. Moreover, $\rightarrow_{\mathtt{flneed}}$ is deterministic.

*Example 15.* Let $t_0 = (\lambda x.(\mathtt{I}(\mathtt{I}x)))\lambda y.y\mathtt{I}$. Needed variable occurrences are highlighted in *orange*.

$$
\begin{array}{ll}
t_0 & \rightarrow_{\mathtt{dB}} (\mathtt{I}(\mathtt{I}x))[x\backslash\lambda y.y\mathtt{I}] \rightarrow_{\mathtt{dB}} x_1[x_1\backslash\mathtt{I}x][x\backslash\lambda y.y\mathtt{I}] \\
& \rightarrow_{\mathtt{dB}} x_1[x_1\backslash x_2[x_2\backslash x]][x\backslash\lambda y.y\mathtt{I}] \rightarrow_{\mathtt{spl}} x_1[x_1\backslash x_2[x_2\backslash x]][x\backslash\!\!\backslash\lambda y.yz_1][z_1\backslash\mathtt{I}] \\
& \rightarrow_{\mathtt{sub}} x_1[x_1\backslash x_2[x_2\backslash\lambda y.yz_1]][x\backslash\!\!\backslash\lambda y.yz_1][z_1\backslash\mathtt{I}] \\
& \rightarrow_{\mathtt{spl}} x_1[x_1\backslash x_2[x_2\backslash\!\!\backslash\lambda y.yz_2][z_2\backslash z_1]][x\backslash\!\!\backslash\lambda y.yz_1][z_1\backslash\mathtt{I}] \\
& \rightarrow_{\mathtt{sub}} x_1[x_1\backslash(\lambda y.yz_2)[x_2\backslash\!\!\backslash\lambda y.yz_2][z_2\backslash z_1]][x\backslash\!\!\backslash\lambda y.yz_1][z_1\backslash\mathtt{I}] \\
& \rightarrow_{\mathtt{spl}} x_1[x_1\backslash\!\!\backslash\lambda y.yz_3][z_3\backslash z_2][x_2\backslash\!\!\backslash\lambda y.yz_2][z_2\backslash z_1][x\backslash\!\!\backslash\lambda y.yz_1][z_1\backslash\mathtt{I}] \\
& \rightarrow_{\mathtt{sub}} (\lambda y.yz_3)[x_1\backslash\!\!\backslash\lambda y.yz_3][z_3\backslash z_2][x_2\backslash\!\!\backslash\lambda y.yz_2][z_2\backslash z_1][x\backslash\!\!\backslash\lambda y.yz_1][z_1\backslash\mathtt{I}]
\end{array}
$$

## 5 A Type System for the $\lambda\mathtt{R}$-calculus

This section introduces a quantitative type system $\mathcal{V}$ for the $\lambda\mathtt{R}$-calculus. Non-idempotent intersection [26] has one main advantage over the idempotent model

[14]: it gives *quantitative* information about the length of reduction sequences to normal forms [21]. Indeed, not only typability and normalization can be proved to be equivalent, but a measure based on type derivations provides an *upper bound* to normalizing reduction sequences. This was extensively investigated in different logical/computational frameworks [5,18,20,25,42,47]. However, no quantitative result based on types exists in the literature for the node replication model, including the attempts done for deep inference [30]. The typing rules of our system are in themselves not surprising (see [46]), but they provide a handy quantitative characterization of fully lazy normalization (Sec. 6).

Types are built on the following grammar of types and multi-types, where $\alpha$ ranges over a set of base types and $\mathtt{a}$ is a special type constant used to type terms reducing to normal abstractions.

$$\textbf{(Types)}\ \sigma := \mathtt{a} \mid \alpha \mid \mathcal{M} \to \sigma \qquad \textbf{(Multi-Types)}\ \mathcal{M} := [\sigma_i]_{i \in I}$$

We write $|\mathcal{M}|$ to denote the **size of a multi-type** $\mathcal{M}$. **Typing contexts**, written $\Gamma$, $\Delta$, $\Sigma$ are functions from variables to multiset types, assigning the empty multiset to all but a finite set of variables. The domain of $\Gamma$ is given by $\mathtt{dom}(\Gamma) := \{x \mid \Gamma(x) \neq [\,]\}$. The **union of contexts**, written $\Gamma + \Delta$, is defined by $(\Gamma + \Delta)(x) := \Gamma(x) \sqcup \Delta(x)$, where $\sqcup$ denotes multiset union. An example is $(x : [\sigma], y : [\tau]) + (x : [\sigma], z : [\tau]) = (x : [\sigma, \sigma], y : [\tau], z : [\tau])$. This notion is extended to several contexts as expected, so that $+_{i \in I} \Gamma_i$ denotes a finite union of contexts, and the empty context when $I = \emptyset$. We write $\Gamma; \Delta$ for $\Gamma + \Delta$ when $\mathtt{dom}(\Gamma) \cap \mathtt{dom}(\Delta) = \emptyset$. **Type judgments** have the form $\Gamma \vdash t : \sigma$, where $\Gamma$ is a typing context, $t$ is a term and $\sigma$ is a type.

$$\frac{}{x : [\sigma] \vdash x : \sigma}\ (\mathtt{ax}) \qquad \frac{\Gamma; x : \mathcal{M} \vdash t : \sigma}{\Gamma \vdash \lambda x.t : \mathcal{M} \to \sigma}\ (\mathtt{abs}) \qquad \frac{\Gamma \vdash t : \mathcal{M} \to \sigma \quad \Delta \vdash u : \mathcal{M}}{\Gamma + \Delta \vdash t\,u : \sigma}\ (\mathtt{app})$$

$$\frac{}{\vdash \lambda x.t : \mathtt{a}}\ (\mathtt{ans}) \qquad \frac{(\Gamma_i \vdash t : \sigma_i)_{i \in I}}{+_{i \in I} \Gamma_i \vdash t : [\sigma_i]_{i \in I}}\ (\mathtt{many}) \qquad \frac{\Gamma; x : \mathcal{M} \vdash t : \sigma \qquad \Delta \vdash u : \mathcal{M}}{\Gamma + \Delta \vdash t[x \triangleleft u] : \sigma}\ (\mathtt{cut})$$

**Fig. 4.** Typing System $\mathcal{V}$

A **(typing) derivation** is a tree obtained by applying the (inductive) typing rules of system $\mathcal{V}$ (Fig. 4), introduced in [46]. The notation $\Phi \rhd \Gamma \vdash t : \sigma$ means there is a derivation named $\Phi$ of the judgment $\Gamma \vdash t : \sigma$ in system $\mathcal{V}$. A term $t$ is typable in system $\mathcal{V}$, or $\mathcal{V}$-typable, iff there is a context $\Gamma$ and a type $\sigma$ such that $\Phi \rhd \Gamma \vdash t : \sigma$. The **size of a type derivation** $\mathtt{sz}(\Phi)$ is defined as the number of its $\mathtt{abs}$, $\mathtt{app}$ and $\mathtt{ans}$ rules. The typing system is **relevant** in the sense that $\Phi \rhd \Gamma \vdash t : \sigma$ implies $\mathtt{dom}(\Gamma) \subseteq \mathtt{fv}(t)$.

Type derivations can be measured by 3-tuples. We use a $+$ operation on 3-tuples as pointwise addition: $(a, b, c) + (e, f, g) = (a + e, b + f, c + g)$. These 3-tuples are computed by a **weighted derivation level** function defined on typing derivations as $\mathsf{D}(\Phi) := \mathsf{M}(\Phi, 1)$, where $\mathsf{M}(-, -)$ is inductively defined below. In the cases $(\mathtt{abs})$, $(\mathtt{app})$ and $(\mathtt{cut})$, we let $\Phi_t$ (resp. $\Phi_u$) be the subderivation of the type of $t$ (resp. $\Phi_u$) and in $(\mathtt{many})$ we let $\Phi_t^i$ be the $i$-th derivation of the type of $t$ for each $i \in I$.

- For $(\mathtt{ax})$, $\mathsf{M}(\Phi_x, m) = (0, 0, 1)$,
- For $(\mathtt{abs})$, $\mathsf{M}(\Phi_{\lambda x.t}, m) = \mathsf{M}(\Phi_t, m) + (1, m, 0)$.
- For $(\mathtt{ans})$, $\mathsf{M}(\Phi_{\lambda x.t}, m) = (1, m, 0)$.
- For $(\mathtt{app})$, $\mathsf{M}(\Phi_{tu}, m) = \mathsf{M}(\Phi_t, m) + \mathsf{M}(\Phi_u, m) + (1, m, 0)$.
- For $(\mathtt{cut})$, $\mathsf{M}\left(\Phi_{t[x \triangleleft u]}, m\right) = \mathsf{M}(\Phi_t, m) + \mathsf{M}(\Phi_u, m + \mathtt{lv}_x(t) + x(\triangleleft))$.
- For $(\mathtt{many})$, $\mathsf{M}(\Phi_t, m) = \sum_{i \in I} \mathsf{M}\left(\Phi_t^i, m\right)$.

Notice that the first and the third components of any 3-tuple $\mathsf{M}(\Phi, m)$ do not depend on $m$. Intuitively, the first (resp. third) component of the 3-tuple counts the number of application/abstraction (resp. $(\mathtt{ax})$) rules in the typing derivation. The second one takes into account the number of application/abstraction rules as well, but *weighted* by the level of the constructor. The 3-tuples are ordered lexicographically.

*Example 16.* Let $\sigma = [\tau] \to \tau$. Consider the following type derivation $\Phi$:

$$\dfrac{\dfrac{}{x : [\tau] \vdash x : \tau}\,(\mathtt{ax}) \qquad \dfrac{\dfrac{\dfrac{}{y : [\sigma] \vdash y : \sigma}\,(\mathtt{ax}) \quad \dfrac{\dfrac{}{z : [\tau] \vdash z : \tau}\,(\mathtt{ax})}{z : [\tau] \vdash z : [\tau]}\,(\mathtt{many})}{y : [\sigma], z : [\tau] \vdash yz : \tau}\,(\mathtt{app})}{y : [\sigma], z : [\tau] \vdash yz : [\tau]}\,(\mathtt{many})}{y : [\sigma], z : [\tau] \vdash x[x \backslash yz] : \tau}\,(\mathtt{cut})$$

This gives $\mathsf{D}(\Phi) = (1, 2, 3)$. Moreover, for $x[x \backslash yz] \to_{\mathtt{app}} (x_1 x_2)[x_1 \backslash y][x_2 \backslash z]$ we have $\Phi' \triangleright y : [\sigma], z : [\tau] \vdash (x_1 x_2)[x_1 \backslash y][x_2 \backslash z] : \tau$ and $\mathsf{D}(\Phi') = (1, 1, 4)$.

## 6 Observational Equivalence

The type system $\mathcal{V}$ characterizes normalization of both `name` and `flneed` strategies as follows: every typable term normalizes and every normalisable term is typable. In this sense, system $\mathcal{V}$ can be seen as a (quantitative) *model* [17] of our call-by-name and call-by-need strategies. We prove these results by studying the appropriate lemmas, notably weighted subject reduction and weighted subject expansion. We then deduce observational equivalence between the `name` and the `flneed` strategies from the fact that their associated normalization properties are both fully characterized by the same typing system.

**Soundness.** Soundness of system $\mathcal{V}$ w.r.t. both $\to_{\mathtt{name}}$ and $\to_{\mathtt{flneed}}$ is investigated in this section. More precisely, we show that typable terms are normalizing for both strategies. In contrast to reducibility techniques needed to show this kind of result for simple types [34], soundness is achieved here by relatively simple combinatorial arguments based again on decreasing measures. We start by studying the interaction between system $\mathcal{V}$ and linear as well as full substitution.

**Lemma 17 (Partial Substitution).** *Let $\Phi \rhd \Gamma; x : \mathcal{M} \vdash \mathtt{C}\langle\langle x\rangle\rangle : \sigma$ and $\sqsubseteq$ denote multiset inclusion. Then, there exists $\mathcal{N} \sqsubseteq \mathcal{M}$ such that for every $\Phi_u \rhd \Delta \vdash u : \mathcal{N}$ we have $\Psi \rhd \Gamma + \Delta; x : \mathcal{M} \setminus \mathcal{N} \vdash \mathtt{C}\langle\langle u\rangle\rangle : \sigma$ and, for every $m \in \mathbb{N}$, $\mathsf{M}(\Psi, m) = \mathsf{M}(\Phi, m) + \mathsf{M}(\Phi_u, m + \mathtt{lv}_\Box(\mathtt{C})) - (0, 0, |\mathcal{N}|)$.*

**Corollary 18 (Substitution).** *If $\Phi_t \rhd \Gamma; x : \mathcal{M} \vdash t : \sigma$ and $\Phi_u \rhd \Delta \vdash u : \mathcal{M}$, then $\Phi \rhd \Gamma + \Delta \vdash t\{x\backslash u\} : \sigma$, and for all $m \in \mathbb{N}$ we have $\mathsf{M}(\Phi, m) \leq \mathsf{M}(\Phi_t, m) + \mathsf{M}(\Phi_u, m + \mathtt{lv}_x(t))$. Moreover, $|\mathcal{M}| > 0$ iff the inequality is strict.*

The key idea to show soundness is that the measure $\mathsf{D}(\_)$ decreases w.r.t. the reduction relations $\rightarrow_{\mathtt{name}}$ and $\rightarrow_{\mathtt{flneed}}$:

**Lemma 19 (Weighted Subject Reduction).** *Let $\Phi_{t_0} \rhd \Gamma \vdash t_0 : \sigma$.*

1. *If $t_0 \rightarrow_\pi t_1$, then there exists $\Phi_{t_1} \rhd \Gamma \vdash t_1 : \sigma$ such that $\mathsf{D}(\Phi_{t_0}) = \mathsf{D}(\Phi_{t_1})$.*
2. *If $t_0 \rightarrow_{\mathtt{s}} t_1$, then there exists $\Phi_{t_1} \rhd \Gamma \vdash t_1 : \sigma$ such that $\mathsf{D}(\Phi_{t_0}) \geq \mathsf{D}(\Phi_{t_1})$.*
3. *If $t_0 \rightarrow_{\mathtt{ndb}} t_1$, then there exists $\Phi_{t_1} \rhd \Gamma \vdash t_1 : \sigma$ such that $\mathsf{D}(\Phi_{t_0}) > \mathsf{D}(\Phi_{t_1})$.*
4. *If $t_0 \rightarrow_{\mathtt{flneed}} t_1$, then there exists $\Phi_{t_1} \rhd \Gamma \vdash t_1 : \sigma$ such that $\mathsf{D}(\Phi_{t_0}) > \mathsf{D}(\Phi_{t_1})$.*

*Proof.* By induction on $\mathtt{r} \in \{\pi, \mathtt{s}, \mathtt{ndb}, \mathtt{flneed}\}$, using Lem. 17 and Cor. 18.

**Theorem 20 (Typability implies name-Normalization).** *Let $\Phi_t \rhd \Gamma \vdash t : \sigma$. Then $t$ is* name*-normalizing.*

*Proof.* Suppose $t$ is not name-normalizing. Since $\rightarrow_{\mathtt{s}}$ is terminating by Cor. 5, then every infinite $\rightarrow_{\mathtt{name}}$-reduction sequence starting at $t$ must necessarily have an infinite number of dB-steps. Moreover, all terms in such an infinite sequence are typed by Lem 19. Therefore, Lem. 19:3 (resp. Lem. 19:2) guarantees that all dB (resp. s) reduction steps involved in such $\rightarrow_{\mathtt{name}}$-reduction sequence strictly decrease (resp. do not increase) the measure $\mathsf{D}(\_)$. This leads to a contradiction because the order $>$ on 3-tuples $\mathsf{D}(\_)$ is well-founded. Then $t$ is necessarily name-normalizing.

**Theorem 21 (Typability implies flneed-Normalization).** *Let $\Phi_t \rhd \Gamma \vdash t : \sigma$. Then $t$ is* flneed*-normalizing. Moreover, $\mathsf{D}(\Phi_t)$ is an upper bound to the length of the* flneed*-reduction evaluation to* flneed*-nf.*

*Proof.* The property trivially holds by Lem. 19:4 since the lexicographic order on 3-tuples is well-founded.

**Completeness.** We address here completeness of system $\mathcal{V}$ with respect to $\rightarrow_{\mathtt{name}}$ and $\rightarrow_{\mathtt{flneed}}$. More precisely, we show that normalizing terms in each strategy are typable. The basic property in showing that consists in guaranteeing that normal forms are typable.

The following lemma makes use of a notion of **needed variable**:
$\mathtt{nv}(x) := \{x\}$, $\mathtt{nv}(tu) := \mathtt{nv}(t)$, $\mathtt{nv}(t[x\backslash\!\backslash u]) := \mathtt{nv}(t)$, $\mathtt{nv}(\lambda x.t) := \emptyset$, $\mathtt{nv}(t[y\backslash u]) := (\mathtt{nv}(t) \setminus \{y\}) \cup \mathtt{nv}(u)$ if $y \in \mathtt{nv}(t)$ and $\mathtt{nv}(t[y\backslash u]) := \mathtt{nv}(t)$ otherwise.

**Lemma 22 (flneed-nfs are Typable).** *Let $t$ be in* flneed*-nf. Then there exists a derivation $\Phi \rhd \Gamma \vdash t : \tau$ such that for any $x \notin \mathtt{nv}(t)$, $\Gamma(x) = [\,]$.*

Because $\texttt{name}$-nfs are also $\texttt{flneed}$-nfs, we infer the following corollary for free.

**Corollary 23 ($\texttt{name}$-nfs are Typable).** *Let $t$ be in $\texttt{name}$-nf. Then there is a derivation $\Phi \rhd \Gamma \vdash t : \tau$.*

Now we need lemmas stating the behavior of partial and full (anti-)substitution w.r.t. typing.

**Lemma 24 (Partial Anti-Substitution).** *Let $\mathtt{C}\langle\langle x\rangle\rangle$, $u$ be terms s.t. $x \notin \mathtt{fv}(u)$ and $\Phi \rhd \Gamma \vdash \mathtt{C}\langle\langle u\rangle\rangle : \sigma$. Then $\exists\Gamma'$, $\exists\Delta$, $\exists\mathcal{M}$, $\exists\Phi'$, $\exists\Phi_u$ s.t. $\Gamma = \Gamma' + \Delta$, $\Phi' \rhd \Gamma' + x : \mathcal{M} \vdash \mathtt{C}\langle\langle x\rangle\rangle : \sigma$ and $\Phi_u \rhd \Delta \vdash u : \mathcal{M}$.*

**Corollary 25 (Anti-Substitution).** *Let $u$ be a term s.t. $x \notin \mathtt{fv}(u)$ and $\Phi \rhd \Gamma \vdash t\{x\backslash u\} : \sigma$. Then $\exists\Gamma'$, $\exists\Delta$, $\exists\mathcal{M}$, $\exists\Phi'$, $\exists\Phi_u$ s.t. $\Gamma = \Gamma' + \Delta$, $\Phi' \rhd \Gamma'; x : \mathcal{M} \vdash t : \sigma$ and $\Phi_u \rhd \Delta \vdash u : \mathcal{M}$.*

To achieve completeness, we show that typing is preserved by anti-reduction. We decompose the property as follows:

**Lemma 26 (Subject Expansion).** *Let $\Phi_{t_1} \rhd \Gamma \vdash t_1 : \sigma$. If $t_0 \to_{\mathtt{r}} t_1$, where $\mathtt{r} \in \{\pi, \mathtt{s}, \boldsymbol{ndb}, \mathtt{flneed}\}$, then there exists $\Phi_{t_0} \rhd \Gamma \vdash t_0 : \sigma$.*

*Proof.* The proof is by induction on $\to_{\mathtt{r}}$ and uses Lem. 24 and Cor. 25.

**Theorem 27 ($\texttt{name}$-Normalization implies Typability).** *Let $t$ be a term. If $t$ is $\texttt{name}$-normalizing, then $t$ is $\mathcal{V}$-typable.*

*Proof.* Let $t$ be $\texttt{name}$-normalizing. Then $t \to^{n}_{\mathtt{name}} u$ and $u$ is a $\texttt{name}$-nf. We reason by induction on $n$. If $n = 0$, then $t = u$ is typable by Cor. 23. Otherwise, we have $t \to_{\mathtt{name}} t' \to^{n-1}_{\mathtt{name}} u$. By the *i.h.* $t'$ is typable and thus by Lem. 26 (because $\to_{\mathtt{ns}}$ is included in $\to_{\mathtt{s}}$), $t$ turns out to be also typable.

**Theorem 28 ($\texttt{flneed}$-Normalization implies Typability).** *Let $t$ be a term. If $t$ is $\texttt{flneed}$-normalizing, then $t$ is $\mathcal{V}$-typable.*

*Proof.* Similar to the previous proof but using Lem. 22 instead of Cor. 23.

Summing up, Thms. 20, 27, 21 and 28 give:

**Theorem 29.** *Let $t$ be a $\lambda\mathtt{R}$-term. $t$ is $\texttt{name}$-normalizing iff $t$ is $\texttt{flneed}$-normalizing iff $t$ is $\mathcal{V}$-typable.*

All the technical tools are now available to conclude observational equivalence between our two evaluation strategies based on node replication. Let $\mathcal{R}$ be any reduction notion on $\Lambda_{\mathtt{R}}$. Then, two terms $t, u \in \Lambda_{\mathtt{R}}$ are said to be **$\mathcal{R}$-observationally equivalent**, written $t \equiv u$, if for any context $\mathtt{C}$, $\mathtt{C}\langle t\rangle$ is $\mathcal{R}$-normalizing *iff* $\mathtt{C}\langle u\rangle$ is $\mathcal{R}$-normalizing.

**Theorem 30.** *For all terms $t, u \in \Lambda_{\mathtt{R}}$, $t$ and $u$ are $\texttt{name}$-observationally equivalent iff $t$ and $u$ are $\texttt{flneed}$-observationally equivalent.*

*Proof.* By Thm. 29, $t \equiv_{\mathtt{name}} u$ means that $\mathtt{C}\langle t\rangle$ is $\mathcal{V}$-typable *iff* $\mathtt{C}\langle u\rangle$ is $\mathcal{V}$-typable, for all $\mathtt{C}$. By the same theorem, this is also equivalent to say that $\mathtt{C}\langle t\rangle$ is $\texttt{flneed}$-normalizing *iff* $\mathtt{C}\langle u\rangle$ is $\texttt{flneed}$-normalizing for any $\mathtt{C}$, *i.e.* $t \equiv_{\mathtt{flneed}} u$.

## 7 Related Works and Conclusion

Several calculi with ES bridge the gap between formal higher-order calculi and concrete implementations of programming languages (see a survey in [40]). The first of such calculi, *e.g.* [1,16], were all based on *structural* substitution, in the sense that the ES operator is syntactically propagated step-by-step through the term structure until a variable is reached, when the substitution finally takes place. The correspondence between ES and Linear Logic Proof-Nets [24] led to the more recent notion of calculi *at a distance* [6,4,2], enlightening a natural and new application of the Curry-Howard interpretation. These calculi implement linear/partial substitution *at a distance*, where the search of variable occurrences is abstracted out with context-based rewriting rules, and thus no ES propagation rules are necessary. A third model was introduced by the seminal work of Gundersen, Heijltjes, and Parigot [33,34], introducing the atomic $\lambda$-calculus to implement node replication.

Inspired by the last approach we introduced the $\lambda\mathtt{R}$-calculus, capturing the essence of node replication. In contrast to [33], we work with an implicit (structural) mechanism of weakening and contraction, a design choice which aims at focusing and highlighting the node replication model, which is the core of our calculus, so that we obtain a rather simple and natural formalism used in particular to specify evaluation strategies. Indeed, besides the proof of the main operational meta-level properties of our calculus (confluence, termination of the substitution calculus, simulations), we use linear and non-linear versions of $\lambda\mathtt{R}$ to specify evaluation strategies based on node replication, namely call-by-name and call-by-need evaluation strategies.

The first description of call-by-need was given by Wadsworth [60], where reduction is performed on *graphs* instead of terms. Weak call-by-need on *terms* was then introduced by Ariola and Felleisen [7], and by Maraist, Odersky and Wadler [54,53]. Reformulations were introduced by Accattoli, Barenbaum and Mazza [3] and by Chang and Felleisen [22]. Our call-by-need strategy is inspired by the calculus in [3], which uses the distance paradigm [6] to gather together meaningful and permutation rules, by clearly separating *multiplicative* from *exponential* rules, in the sense of Linear Logic [27].

Full laziness has been formalized in different ways. Pointer graphs [60,59] are DAGs allowing for an elegant representation of sharing. Labeled calculi [15] implement pointer graphs by adding annotations to $\lambda$-terms, which makes the syntax more difficult to handle. Lambda-lifting [38,39] implements full laziness by resorting to translations from $\lambda$-terms to supercombinators. In contrast to all the previous formalisms, our calculus is defined on standard $\lambda$-terms with explicit cuts, without the use of any complementary syntactical tool. So is Ariola and Felleisen's call-by-need [7], however, their notion of full laziness relies on external (ad-hoc) meta-level operations used to extract the skeleton. Our specification of call-by-need enjoys fully lazy sharing, where the skeleton extraction operation is internally encoded in the term calculus operational semantics. Last but not least, our calculus has strong links with proof-theory, notably deep inference.

Balabonski [10,9] relates many formalisms of full laziness and shows that they are equivalent when considering the number of $\beta$-steps to a normal form. It would then be interesting to understand if his unified approach, (abstractly) stated by means of the theory of residuals [50,51], applies to our own strategy.

We have also studied the calculus from a semantical point of view, by means of intersection types. Indeed, the type system can be seen as a model of our implementations of call-by-name and call-by-need, in the sense that typability and normalization turn out to be equivalent.

Intersection types go back to [23] and have been used to provide characterizations of qualitative [14] as well as quantitative [21] models of the $\lambda$-calculus, where typability and normalization coincide. Quantitative models specified by means of non-idempotent types [26,48] were first applied to the $\lambda$-calculus (see a survey in [19]) and to several other formalisms ever since, such as call-by-value [25,20], call-by-need [42,5], call-by-push-value [31,18] and classical logic [47]. In the present work, we achieve for the first time a quantitative characterization of fully lazy normalization, which provides upper bounds for the length of reduction sequences to normal forms.

The characterizations provided by intersection type systems sometimes lead to observational equivalence results (*e.g.* [42]). In this work we succeed to prove observational equivalence related to a fully lazy implementation of weak call-by-need, a result which would be extremely involved to prove by means of syntactical tools of rewriting, as done for weak call-by-need in [7]. Moreover, our result implies that our node replication implementation of full laziness is observationally equivalent to standard call-by-name and to weak call-by-need (see [42]), as well as to the more semantical notion of neededness (see [45]).

A Curry-Howard interpretation of the logical *switch* rule of deep inference is given in [58,57] as an end-of-scope operator, thus introducing the *spinal atomic $\lambda$-calculus*. The calculus implements a refined optimization of call-by-need, where only the *spine* of the abstraction (tighter than the skeleton) is duplicated. It would be interesting to adapt the $\lambda\mathtt{R}$-calculus to spine duplication by means of an appropriate end-of-scope operator, such as the one in [37]. Further optimizations might also be considered.

Finally, this paper only considers weak evaluation strategies, *i.e.* with reductions forbidden under abstractions, but it would be interesting to extend our notions to full (strong) evaluations too [29,12]. Extending full laziness to classical logic would be another interesting research direction, possibly taking preliminary ideas from [36]. We would also like to investigate (quantitative) *tight* types for our fully lazy strategy, as done for weak call-by-need in [5], which does not seem evident in our node replication framework.
